# Stuttering Cured

**Published:** 1891-06

**Authors:** 


### STUTTERING CURED.

All stutterers are of the nervous temperament, and in their haste to speak so much nervous energy is sent to the vocal organs that they cannot act fast enough for it to escape, and confusion is the result. All that is necessary is to divert a part of that energy to some other operation—that is, to divert the mind partly to some other thing; thus, at the enunciation of every syllable tap any part of the body with the finger, or the floor with the foot, or wink the eye, and the person will cease to stutter on the instant. Persist in the practice, and the cure will be permanent.

HERBA VITA.—Read the advertisement of this simple remedy. We have proved its value and fully indorse all that is said of it.

